# The Relationship of Body Mass Index and Blood Pressure with Corneal Biomechanical Parameters in Healthy Subjects

**Published:** 2017

**Authors:** Mohamad-Reza SEDAGHAT, Farshad ASKARIZADEH, Mohsen NEMATY, Foroozan NAROOIE-NOORI, Javad HERAVIAN, Tahereh RAKHSHANDADI, Sattar RAJABI

**Affiliations:** 1 Cornea Research Center, Khatam‐Al‐Anbia Hospital, Mashhad University of Medical Sciences, Mashhad, Iran; 2 Department of Optometry, Refractive Errors Research Center, School of Paramedical Sciences, Mashhad University of Medical Sciences, Mashhad, Iran; 3 Department of Nutrition, School of medicine, Mashhad University of Medical Sciences, Mashhad, Iran; 4 Department of Optometry, school of rehabilitation sciences, Zahedan University of Medical Sciences, Zahedan, Iran

**Keywords:** Cornea, Body Mass Index, Blood Pressure, Healthy Subjects

## Abstract

This study aimed to assess the possible relationship of body mass index (BMI) and blood pressure (BP) with corneal biomechanical parameters in healthy subjects. The study included 88 eyes of 88 healthy subjects aged 20–40 years. After a thorough medical history, a digital sphygmomanometer was used to measure the systolic blood pressure (SBP) and diastolic blood pressure (DBP). In addition, several hematological and biochemical parameters were determined to assess general health. Prior the ophthalmic examination, the body height and weight were measured; then, the BMI was calculated. Finally, after comprehensive ophthalmic examination, all cases were evaluated with Pentacam (Oculus) in order to rule out corneal ectasia; then, the corneal biomechanical parameters of all individuals were measured using the Scheimpflug-based Corvis ST (Oculus Optikgeräte GmbH, Wetzlar, Germany). If the measurements of the hematological and biochemical parameters were within normal range, the results of the Corvis ST, BMI, and BP were included in the analysis carried out with SPSS software. The mean (± standard deviation [SD]) BMI, SBP, DBP, intraocular pressure (IOP), central corneal thickness (CCT), deformation amplitude, radius, and peak distance was 27.24 ± 4.80 kg/m2, 116.47 ± 11.21 mmHg, 80.51 ± 5.68 mmHg, 15.10 ± 1.70 mmHg, 533.10 ± 30.97 micrometer, 1.03 ± 0.11 mm, 7.51 ± 0.86 mm, and 5.03 ± 0.30 mm, respectively. Results showed no significant difference in IOP, CCT, peak distance, radius, and deformation amplitude between different BMI subcategories defined by World Health Organization (all P > 0.05). The results of the Corvis ST showed that corneal biomechanical parameters had no significant correlation with BMI, SBP, and DBP in three subgroups of BMI and all participants (all P > 0.05) but the results showed a positive correlation between CCT and IOP (P < 0.001, r = 0.504) in all participants. CCT and IOP had no correlation with BMI, SBP, and DBP (P > 0.05). This study showed that BMI and BP had no correlation with corneal biomechanical parameters in healthy subjects using the Corvis ST.

## INTRODUCTION

Corneal biomechanics studies equilibrium of the cornea under the application of any force [1]. As a viscoelastic material, the cornea relies on the biochemical nature and physical structure of its basic components and their relative quantities [2]. Some devices and methods are used to evaluate different corneal biomechanical parameters [2, 3]. The first device introduced for in vivo measurement of corneal biomechanics was the Ocular Response Analyzer (ORA; Reichert Ophthalmic Instruments, Inc., Buffalo, NY, USA), which is used to determine corneal hysteresis (CH) and corneal resistance factor (CRF) in the clinical setting [2, 4]. The Scheimpflug-based corneal visualization technology (Oculus Optikgeräte GmbH, Wetzlar, Germany) has been recently developed to evaluate corneal biomechanics. This instrument shows the cornea’s dynamic deformation and records the deformation parameters [2, 5]. Corneal biomechanical parameters are necessary for preoperative evaluation of refractive surgery candidates, accurate intraocular pressure (IOP) determination, and proper diagnosis or monitoring of ocular diseases such as keratoconus and glaucoma [5, 6]. The body mass index (BMI) is a surrogate of the balance between an individual’s height and weight, which is calculated as weight in kilograms divided by the squared height in meters (kg/m^2^) [7]. The World Health Organization (WHO) has classified the BMI into four subcategories [8]. A large body of literature suggests that some ocular conditions such as cataract [9], glaucoma [10], diabetic retinopathy [11], and age-related macular degeneration [12] have a potential association with different BMI categories. Some studies have reported a positive association between BMI and blood pressure (BP) in the healthy population [13, 14]. Furthermore, there is a strong relationship between BMI and central corneal thickness (CCT) [15]. Fontes et al. demonstrated that corneal biomechanical parameters, including CH and CRF, are positively correlated with CCT [6]. Moreover, Leung et al. reported that a greater CCT is associated with smaller corneal deformation amplitude [16]. Since BMI is associated with BP, and CCT is correlated with BMI and corneal biomechanics, we hypothesized that corneal biomechanical properties may be related to BMI. The aim of this study was to evaluate the relationship of corneal biomechanical properties, measured using the Corvis ST, with BMI and BP in healthy subjects. To our knowledge, this is the first attempt to investigate the association of corneal biomechanics with BMI and BP by this specific methodology.

## MATERIALS AND METHODS

Eighty-eight healthy subjects aged 20–40 years were enrolled in this prospective study in June 2015. The Institutional Review Board and the Ethics Committee of Mashhad University of Medical Sciences approved the study and ensured its protocol followed the tenets of the Declaration of Helsinki (Code Number: 940149). All selected subjects received information about the study and written informed consent was obtained from them. The study was performed in Mashhad, northeast of Iran, and all cases were residents of Mashhad and were Iranian in origin, with the same ethnicity. Before starting the ophthalmic examination, a full medical history was taken and all participants underwent medical assessments including BP measurement and hematological and biochemical analysis. Using a digital sphygmomanometer (BP AG1-20, Microlife, Widnau, Switzerland), BP was measured after a 5-minute rest by a single experienced physician who was blinded to the objectives of the study. We recorded the mean value of three successive readings taken at 2-minute intervals between 10:00 AM and 12:00 AM. A normal BP was described as a systolic blood pressure (SBP) <120 mmHg and a diastolic blood pressure (DBP) <80 mmHg [13]. Specifically, we excluded the volunteers with an abnormal SBP and DBP, so SBP and DBP were within the normal range of BP (SBP <120 mmHg and DBP <80 mmHg) in all participants [13]. Hematological and biochemical analysis was performed on the day after BP measurement. Venous blood samples were collected after at least 15 minutes sitting at rest or in the supine position by a single experienced physician between 8:00 AM and 10:00 AM in the morning after an overnight fast. Then, hematological (hemoglobin, hematocrit, platelets, white blood cells, red blood cells (red blood cell indices: mean red cell volume, mean corpuscular hemoglobin, mean corpuscular hemoglobin concentration)) and biochemical parameters (fast blood sugar, urea, creatinine, uric acid, albumin, selenium, sodium, potassium, phosphor, calcium, iron, zinc, cholesterol, triglyceride, low-density lipoprotein cholesterol [LDL-C], high-density lipoprotein cholesterol [HDL-C], LDL/HDL ratio) were analyzed.

The method used for hematological and biochemical measurements and analyses has been described in the published literature [17]. The participants were systematically assessed by an experienced physician and patients with clinical manifestations of systemic diseases and abnormal hematological and biochemical measurements were excluded from the study. The participants’ height (in meters) and weight (in kilograms) were measured using a wall-mounted metric ruler and a digital scale, respectively. The measurements were done in the standing position with minimal clothing and no shoes. The BMI was determined and categorized using the WHO classification, which is the same for all age groups and both sexes. The BMI subgroups include <18.5 kg/m^2^ (underweight), 18.5–24.9 kg/m^2^ (normal weight), 25.0–29.9 kg/m^2^ (overweight), and ≥30.0 kg/m^2^ (obese) [8]. After a thorough medical evaluation and ocular history taking, all participants underwent a comprehensive ophthalmic examination, including measurement of uncorrected distance visual acuity (UDVA) and corrected distance visual acuity (CDVA) with a high-contrast visual acuity chart, manifest (non-cycloplegic) and cycloplegic refraction (Topcon KR-1, Topcon, Tokyo, Japan), regularity status of the retinoscopic reflex, non-contact computerized tonometry (Topcon CT-1/CT-1P, Topcon), slit-lamp biomicroscopy, dilated fundoscopy, and Scheimpflug-based tomography (Pentacam HR, Oculus, Optikgeräte GmbH, Wetzlar, Germany). The study group comprised healthy individuals with normal eyes, a UDVA or CDVA of 0.00 logarithm of the minimum angle of resolution (LogMAR) or higher (Snellen equivalent 20/20 or better), and normal tomography (Pentacam HR). Ocular evaluations and ophthalmic examinations were done by an experienced ophthalmologist and an optometrist. We excluded patients with underlying systemic diseases, connective tissue disorders, a history of corneal and/or intraocular surgery or injury, ocular hypertension, glaucoma, high refractive errors, corneal scar, or corneal pathologies potentially affecting the measurement of corneal biomechanics, smokers, those who were pregnant or lactating, and those who consumed certain drugs. Soft contact lenses were removed at least 2 weeks before these measurements. None of the subjects used hard contact lenses. 

We selected participants with low refractive errors (spherical equivalent between plano and ± 3.00 D and astigmatism <1.00 D on cycloplegic refraction) to rule out the effect of high refractive errors on ocular parameters [18].

The Scheimpflug-based Corvis ST was employed for measuring corneal biomechanical parameters; this device is used for noninvasive imaging of the cornea’s dynamic deformation reaction to a puff of air. The mechanism of the Corvis ST has been already described in other studies [19-22]. The Corvis ST outputs are IOP, CCT, central radius of curvature at the highest concavity, distance of the two surrounding peaks of the cornea at the maximum concavity (peak distance), and supreme deformation amplitude defined as the corneal apex movement from the beginning of deformation to the maximum concavity [21]. In addition to the mentioned biomechanical parameters, the Corvis ST has many other outputs [22] but these sophisticated calculations cannot be considered as pure biomechanical parameters. Measurements using the Corvis ST were carried out three times per individual and the average of the three measurements was used in the statistical analyses. A 5-minute interval was considered between the measurements. The repeatability and reproducibility of the Corvis ST for measuring corneal biomechanical parameters have been described previously [23]. All the devices used in this investigation were calibrated by the manufacturer’s representative before the study, and all the ophthalmic imaging and measurements were performed in a consistent manner based on the manufacturer’s instruction by a single experienced optometrist who was blinded to the objectives of the study. High quality measurements were accepted and erroneous readings were repeated after 5 minutes. All general assessments, ophthalmic evaluations, corneal imaging studies, and hematological and biochemical measurements were carried out in six consecutive days. Finally, the data of only one eye were selected randomly for statistical analysis.

The normal distribution of the parameters was assessed using the Kolmogorov–Smirnov test in SPSS software (version 22; SPSS, Inc., Chicago, IL). One-way analysis of variance (ANOVA) was used to compare the parameters with a normal distribution and a post-hoc Tukey’s test was performed for variables with a statistically significant difference according to one-way ANOVA. The Kruskal–Wallis test was used to compare non-parametric parameters. Pearson’s correlation coefficient was used to evaluate the correlations between parameters. In addition to statistical analysis, the possible effect of sex was considered and all analyses were conducted separately for men and women. P-values less than 0.05 were considered significant. 

## RESULTS

We investigated 88 eyes of healthy subjects (51 [57.95%] men and 37 [42.05%] women). The mean (± standard deviation [SD]) age, spherical equivalent, and visual acuity (UDVA or CDVA) of the participants was 34 ± 9.13 years, -0.58 ± 1.21 D, and 0.017 ± 0.06 LogMAR, respectively. Moreover, the mean (± SD) BMI, SBP, DBP, IOP, CCT, deformation amplitude, curvature radius, and peak distance was 27.24 ± 4.80 kg/m^2^, 116.47 ± 11.21 mmHg, 80.51 ± 5.68 mmHg, 15.10 ± 1.70 mmHg, 533.10 ± 30.97 m, 1.03 ± 0.11 mm, 7.51 ± 0.86 mm, and 5.03 ± 0.30 mm, respectively. Considering the WHO categorization for BMI, none of the subjects had a BMI <18.5 kg/m^2^ (underweight), so we classified our participants into three groups: group 1 (normal weight), group 2 (overweight), and group 3 (obese) [8]. Data for sex, age, BMI, IOP, CCT, SBP, and DBP of the subjects in different BMI subgroups are presented in Table 1. The results showed that the majority of study samples (39 cases) were in group 2 (overweight) and the mean age of this group (38.80 ± 9.44 years) was higher than that of others. Also, SBP and DBP were highest in group 3 (121.50 ± 11.82 mmHg and 83.25 ± 4.37 mmHg, respectively).

**Table 1:** Demographic and Clinical Data for each Body Mass Index (BMI) Group: Group 1 (BMI: 18.5–24.9 kg/m^2 ^[Normal Weight]), Group 2 (BMI: 25.0–29.9 kg/m^2 ^[Overweight]), and Group 3 (BMI ≥ 30.0 kg/m^2 ^[Obese])

	Group 1 (mean ± SD)	Group 2 (mean ± SD)	Group 3 (mean ± SD)	P-value
Number	29	39	20	
Sex	16 (55.17%) females	13 (33.33%) females	8 (40%) females	
Age				
** All participants**	30.10 ± 7.50	38.80 ± 9.44	35.15 ± 7.13	<0.001*
** Males**	30.70 ± 7.41	38.65 ± 9.52	33.92 ± 6.60	0.022 *
** Females**	29.62 ± 7.78	39.08 ± 9.64	37.00 ± 7.95	0.015 *
BMI				
** All participants**	22.27 ± 1.64	27.60 ± 1.46	33.81 ± 3.70	<0.001 *
** Males**	22.58 ± 1.58	27.35 ± 1.42	34.07 ± 3.50	<0.001 *
** Females**	22.02 ± 1.70	28.00 ± 1.50	33.42 ± 4.22	<0.001 *
IOP^║^				
** All participants**	15.35 ± 2.04	14.74 ± 1.45	15.45 ± 1.56	0.207 *
** Males**	15.19 ± 1.45	14.81 ± 1.54	15.42 ± 1.68	0.556 *
** Females**	15.47 ± 2.45	14.62 ± 1.29	15.50 ± 1.49	0.481 *
CCT^║^				
** All participants**	540.69 ± 29.16	528.64 ± 30.69	530.80 ± 33.45	0.267 *
** Males**	544.92 ± 28.62	530.31 ± 32.88	525.08 ± 27.87	0.239 *
** Females**	537.25 ± 30.06	525.31 ± 26.70	539.38 ± 40.95	0.730 *
SBP				
** All participants**	111.73 ± 10.38	117.57 ± 10.38	121.50 ± 11.82	0.011^Ɨ^
** Males**	117.69 ± 10.13	118.46 ± 10.37	122.08 ± 9.41	0.500 ^Ɨ^
** Females**	106.88 ± 7.93	115.77 ± 10.58	120.63 ± 15.46	0.008 ^Ɨ^
DBP				
** All participants**	78.10 ± 5.25	80.90 ± 6.06	83.25 ± 4.37	0.008 ^Ɨ^
** Males**	79.62 ± 03.20	80.96 ± 5.30	83.33 ± 4.44	0.140 ^Ɨ^
** Females**	76.88 ± 6.29	80.77 ± 7.60	83.13 ± 4.58	0.066 ^Ɨ^

**BMI = body mass index (kg/m** ^2^ **), IOP = intraocular pressure (mmHg), CCT = central corneal thickness (m), SBP = systolic blood pressure (mmHg), DBP = diastolic blood pressure (mmHg).**

There were no missing data. A P-value < 0.05 was considered statistically significant.

* one-way ANOVA test,

Ɨ Kruskal–Wallis test.

║ measured by Corvis ST.

A post hoc Tukey’s test for age showed significant differences between group 1 and 2 (P < 0.001), 1 and 3 (P = 0.030), and 2 and 3 (P < 0.001). In addition, a post hoc Tukey’s test for BMI showed significant differences between group 1 and 2, 1 and 3, and 2 and 3 (P < 0.001 for all the comparisons). The results of peak distance, radius, and deformation amplitude of the subjects in different BMI groups using the Corvis ST are shown in Table 2. According to our findings, the values for deformation amplitude in group 3 were lower (1.00 ± 0.11) than those of other groups (group 1: 1.03 ± 0.13, group 2: 1.05 ± 0.10). In addition, there were no significant differences for peak distance and radius between the three BMI groups (P = 0.613 and P = 0.351, respectively). The results of Pearson’s test regarding the correlation of corneal biomechanical parameters with BMI, SBP, and DBP are presented in Table 3. The results of the Corvis ST showed that corneal biomechanical parameters had no significant correlation with BMI, SBP, and DBP in the three groups of BMI and in all participants (all P > 0.05).

**Table 2:** Comparison of Corvis ST-measured Corneal Biomechanical Parameters between Body Mass Index (BMI) Groups: Group 1 (BMI: 18.5–24.9 kg/m^2 ^[Normal Weight]), Group 2 (BMI: 25.0–29.9 kg/m^2 ^[Overweight]), and Group 3 (BMI ≥ 30.0 kg/m^2 ^[Obese])

	Group 1		Group 2	Group 3		P-value
Peak distance						
** All participants**	5.03 ± 0.40		5.06 ± 0.26	4.98 ± 0.22		0.613*
** Males**	5.15 ± 0.39		5.10 ± 0.25	4.98 ± 0.22		0.337 *
** Females**	4.93 ± 0.39		5.00 ± 0.28	4.98 ± 0.23		0.827 *
Radius						
** All participants**	7.67 ± 0.92		7.37 ± 0.76	7.56 ± 0.96		0.351 *
** Males**	7.70 ± 0.91		7.23 ± 0.78	7.30 ± 0.82		0.245
** Females**	7.64 ± 0.96		7.64 ± 0.64	7.98 ± 1.06		0.742 *
Deformation Amplitude						
** All participants**	1.03 ± 0.13		1.05 ± 0.10	1.00 ± 0.11		0.374 *
** Males**	1.06 ± 0.12		1.05 ± 0.10	1.01 ± 0.11		0.478 *
** Females**	1.01 ± 0.14		1.04 ± 0.10	0.99 ± 0.10		0.692 *

Peak distance (mm), radius (mm), deformation amplitude (mm).

* one-way ANOVA test. A P-value < 0.05 was considered statistically significant. There were no missing data.

**Table 3:** Correlation of Corvis ST-measured Corneal Biomechanical Parameters with Body Mass Index (BMI), Systolic Blood Pressure, and Diastolic Blood Pressure. The Three BMI Groups were: Group 1 (BMI: 18.5–24.9 kg/m^2^ [Normal Weight]), Group 2 (BMI: 25.0–29.9 kg/m^2^ [Overweight]), and Group 3 (BMI ≥ 30.0 kg/m^2^ [Obese])

	Peak distance		Radius		Deformation amplitude	
	**p-value**	**r** *	**P-value**	r*	**P-value**	**r** *
All Participants						
**BMI**	0.432	-0.085	0.814	-0.025	0.252	-0.123
**SBP**	0.708	0.040	0.846	-0.021	0.997	0.000
**DBP**	0.062	-0.200	0.971	-0.004	0.372	-0.096
Group 1						
**BMI **	0.134	-0.285	0.888	0.027	0.138	-0.282
**SBP **	0.380	0.169	0.669	0.083	0.302	0.199
**DBP **	0.115	-0.299	0.378	0.170	0.203	-0.243
Group 2						
**BMI **	0.090	-0.275	0.842	-0.033	0.412	-0.135
**SBP **	0.219	-0.201	0.632	0.079	0.055	-0.310
**DBP **	0.180	-0.219	0.934	0.014	0.0476	-0.118
Group 3						
**BMI **	0.211	0.292	0.588	0.129	0.854	0.044
**SBP **	0.146	0.338	0.660	-0.105	0.172	0.318
**DBP **	0.543	0.145	0.373	-0.210	0.093	0.386

Peak distance (mm), radius (mm), deformation amplitude (mm), BMI = body mass index (kg/m^2^), SBP = systolic blood pressure (mmHg), DBP = diastolic blood pressure (mmHg).

* Pearson’s correlation coefficient. There were no missing data.

There was a positive correlation between CCT and IOP (P < 0.001, r = 0.504) in all participants. Also, our results revealed a statistically significant correlation between CCT and IOP in group 1 (P = 0.001, r = 0.605) and group 3 (P = 0.001, r = 0.691). Meanwhile, our results showed a weak correlation between CCT and IOP in group 2 (P = 0.048, r = 0.306). In addition, no correlation was found between BMI and SBP in group 1 (P = 0.445, r = 0.148) and group 2 (P = 0.337, r = 0.146) but BMI was found to be associated with SBP in group 3 (P = 0.015, r = 0.534). Also, the correlation between BMI and DBP was not statistically significant in different BMI groups (group 1 [P = 0.265, r = 0.214], group 2 [P = 0.260, r = 0.185], and group 3 [P = 0.954, r = 0.014]). The BMI of all healthy subjects had a statistically significant correlation with SBP (P < 0.001, r = 0.418) and DBP (P = 0.001, r = 0.347). The correlation of IOP and CCT with BMI, SBP, and DBP in different BMI groups is presented in Table 4. CCT and IOP had no correlation with BMI, SBP, and DBP (all P > 0.05). 

**Table 4:** Correlation of Body Mass Index (BMI), systolic Blood Pressure, and Diastolic Blood Pressure with Intraocular Pressure and Central Corneal Thickness in BMI Groups: Group 1 (BMI: 18.5–24.9 kg/m^2^ [Normal Weight]), Group 2 (BMI: 25.0–29.9 kg/m^2^ [Overweight]), and Group 3 (BMI ≥ 30.0 kg/m^2^ [Obese])

	**IOP**		**CCT**	
	P-value	r*	P-value	r*
All participants				
** BMI**	0.401	0.091	0.629	-0.052
** SBP**	0.994	-0.001	0.538	0.066
** DBP**	0.699	0.042	0.874	-0.017
Group 1				
** BMI**	0.046	0.373	0.432	0.152
** SBP**	0.649	-0.088	0.264	0.214
** DBP**	0.285	0.206	0.069	0.343
Group 2				
** BMI**	0.438	0.128	0.967	0.007
** SBP**	0.117	0.255	0.855	-0.045
** DBP**	0.730	0.057	0.113	-0.366
Group 3				
** BMI**	0.619	0.118	0.308	0.240
** SBP**	0.328	-0.231	0.317	0.164
** DBP**	0.167	-0.321	0.995	0.001

BMI = body mass index (kg/m^2^μ

* Pearson’s correlation coefficient. There were no missing data.

## DISCUSSION

The relationship between BMI and BP has been assessed in the published literature [13, 14] and these two parameters have a significant correlation with IOP [24, 25]. In addition, the association of CCT with BMI [15], IOP [25], and corneal biomechanics [6, 16] has already been documented. Considering the mentioned correlations and the significant role of corneal biomechanics [5], this study was designed to investigate the possible association of corneal biomechanical metrics with BMI and BP. The results revealed no significant differences in IOP, CCT, peak distance, radius, and deformation amplitude between different BMI subcategories. Furthermore, corneal biomechanical parameters measured with the Corvis ST in healthy subjects showed no correlation with BMI, SBP, and DBP in different BMI subcategories. As for BMI groups 2 and 3, IOP and CCT had no correlation with BMI, SBP, and DBP. Meanwhile, there was a weak correlation between BMI and IOP in group 1. We concluded a significant positive correlation between CCT and IOP in all participants and BMI subgroups. In addition, the BMI of all healthy subjects had an association with SBP and DBP but our findings showed no association between BMI and SBP in groups 1 and 2. The correlation between BMI and SBP in group 3 was positive but not strong. Notably, our findings suggested no correlation between BMI and DBP in different BMI groups. It is worth mentioning that this finding can result from the effect of sample size in the study groups. Our findings confirm the results of previous studies such as the ones conducted by Karadag et al. and Albuquerque et al. in which no correlation was found between BMI and IOP [7, 26]. Karadag et al. showed no difference in IOP between BMI subcategories using the Pascal dynamic contour tonometer, and reported no difference in SBP and DBP between three BMI subcategories [7]. Although Karadag et al. studied healthy subjects, they did not perform hematology or biochemistry analysis. On the other hand, Albuquerque et al. reported their results for BMI and IOP in children [26] but our participants were in the age range of 20–40 years. Cohen et al. showed a positive linear correlation between BMI and IOP in both men and women [24]. Our findings contradict the results of the mentioned study since we found no correlation between BMI and IOP. Cohen et al. conducted a retrospective study and used the Goldmann applanation tonometry [24] but our study was prospective and we used the Scheimpflug-based noninvasive air puff system. In another investigation, George et al. reported a statistically significant positive correlation between BMI and IOP and also between BMI and BP in an overweight and obese Nigerian population [8]. Their results in an overweight and obese population are in contrast to our findings. However, their results did not show any correlation between BMI and BP in normal weight subjects [8], which is in accordance with our findings. The differences in the results for overweight and obese participants may be related to the method of selecting healthy subjects as well as other epidemiological factors. As for the relationship between CCT and BMI, Elflein et al. reported that CCT was associated with BMI in an adult white cohort in a population-based study [15]. The diversity of the findings in these studies can be related to inclusion and exclusion criteria. Elflein et al. investigated CCT and BMI without excluding participants with diabetes and dyslipidemia. In the field of corneal biomechanics, a few studies have evaluated corneal biomechanical parameters in different ocular and systemic conditions but no study has investigated the correlation of corneal biomechanics with BMI and BP simultaneously.

The high and ever-increasing prevalence of overweight as well as obesity and hypertension is a huge public health challenge. The adverse consequences of these abnormalities can potentially affect the public health and can be considered as risk factors for systemic disorders [27, 28]. Considering the significant role of corneal biomechanics in different ocular and systemic conditions [1, 5], assessment of the correlation of BMI and BP with corneal biomechanics may be clinically useful; for example, many patients with different BMI and BP values undergo corneal refractive surgery. The results of this study showed that BMI and normal BP in healthy subjects are not correlated with corneal biomechanical parameters measured using the Corvis ST. 

Based on mechanistic reasoning philosophy [29], the inductive inference for the association of BMI with corneal biomechanical parameters may seem rational, but our study showed that probabilistic proponents and mechanistic reasoning in this case of evidence-based medicine can lead us astray. The major specification of the present study was its healthy subjects which Corvis ST showed that corneal biomechanical parameters had no significant correlation with BMI, SBP, and DBP
